# Mycofactocin and the mycobacterial electron transport chain

**DOI:** 10.7554/eLife.106286

**Published:** 2025-03-05

**Authors:** Stephanie M Stuteley, Ghader Bashiri

**Affiliations:** 1 Laboratory of Microbial Biochemistry and Biotechnology, School of Biological Sciences, The University of Auckland Auckland New Zealand

**Keywords:** *Mycolicibacterium smegmatis*, mycofactocin, mycobacteria, respiration, redox cofactor, oxidoreductase, Other

## Abstract

In the bacterium *M. smegmatis*, an enzyme called MftG allows the cofactor mycofactocin to transfer electrons released during ethanol metabolism to the electron transport chain.

**Related research article** Graça AP, Nikitushkin V, Ellerhorst M, Vilhena C, Klassert TE, Starick A, Siemers M, Al-Jammal WK, Vilotijevic I, Slevogt H, Papenfort K, Lackner G. 2025. MftG is crucial for ethanol metabolism of mycobacteria by linking mycofactocin oxidation to respiration. *eLife* **13**:RP97559. doi: 10.7554/eLife.97559.

To survive, bacteria must adapt to ever-changing environments and, as a result, have developed diverse strategies to respond to this challenge. In particular, species from the Mycobacterium genus can flexibly adjust their metabolism (Cook et al., 2009), allowing them to break down a wide range of nutrients to produce energy (de Carvalho et al., 2010).

This metabolic flexibility, displayed by both environmental and pathogenic species, is partly due to a variety of unusual cofactors – molecules that help enzymes to catalyse reactions (Peña-Ortiz et al., 2020). Among these is mycofactocin, a peptide-derived cofactor that has been linked to the metabolism of cholesterol and fatty acids in *M. tuberculosis* (Krishnamoorthy et al., 2019; Mendauletova and Latham, 2022) and ethanol in *M. smegmatis*. Previous work showed that an enzyme known as Mdo/Mno, which breaks down alcohol and uses mycofactocin as a cofactor, is essential for *M. smegmatis* to consume ethanol (Krishnamoorthy et al., 2019). Now, in eLife, Gerald Lackner and colleagues – including Ana Patrícia Graça as first author – report that by carrying electrons released when ethanol is broken down, mycofactocin links ethanol metabolism to the generation of cellular energy in the form of ATP (Graça et al., 2025).

Previous studies have shown that multiple enzymes encoded in a gene cluster known as *mft* contribute to the production of bioactive mycofactocin molecules (Ayikpoe et al., 2018; Haft, 2011). However, one gene in the cluster, which encodes an enzyme known as MftG, remained uncharacterised. Graça et al. (who are based at the Leibniz Institute for Natural Products and Infection Biology, the Hans Knöll Institute and various other research institutes in Germany) began by deleting the gene encoding MftG in *M. smegmatis*. When provided with ethanol as a nutrient source, strains lacking this gene showed impaired growth, displaying features of mild starvation, such as disrupted cell division and energy production.

Analysis of gene expression in cells lacking the MftG gene revealed that processes related to cell division, including DNA replication and peptidoglycan biosynthesis, were downregulated. Additionally, cellular respiratory machinery had been remodelled to compensate for a shortage of electron donors. These observations highlight the modified bacterial strain’s ability to compensate for impaired mycofactocin metabolism.

Graça et al. next investigated how deleting the gene for MftG leads to starvation-like features. Measuring bacterial respiration showed that cells lacking MftG consumed only around 45% of the oxygen consumed by the parent cells. This indicates that fewer electrons were being delivered to the electron transport chain, the final stage of energy production, where they are ultimately accepted by oxygen.

Analysing the contents of the cells using mass spectrometry revealed that strains lacking MftG accumulated higher levels of reduced mycofactocin (those that have gained electrons) while having almost no detectable oxidised mycofactocin (those that have lost electrons). In contrast, a strain containing an additional copy of the MftG gene showed a substantial increase in oxidised mycofactocin. These findings indicate that MftG catalyses the oxidation of mycofactocin. Additionally, Graça et al. suggest that the oxidation of mycofactocin, which allows it to be recycled, is the rate-limiting step in ethanol metabolism.

This prompted the team to investigate whether MftG and mycofactocin could transfer electrons to the electron transport chain, which is embedded in the mycobacterial membrane. While known electron donors to the electron transport chain, such as NADH and succinate, were able to contribute to the production of energy in *M. smegmatis* membrane preparations when MftG was absent, reduced mycofactocin could only do so when MftG was present. This indicates that MftG facilitates electron transfer from reduced mycofactocin to the electron transport chain, thereby directly linking the electrons generated during ethanol metabolism to energy production (Figure 1).

**Figure 1.**
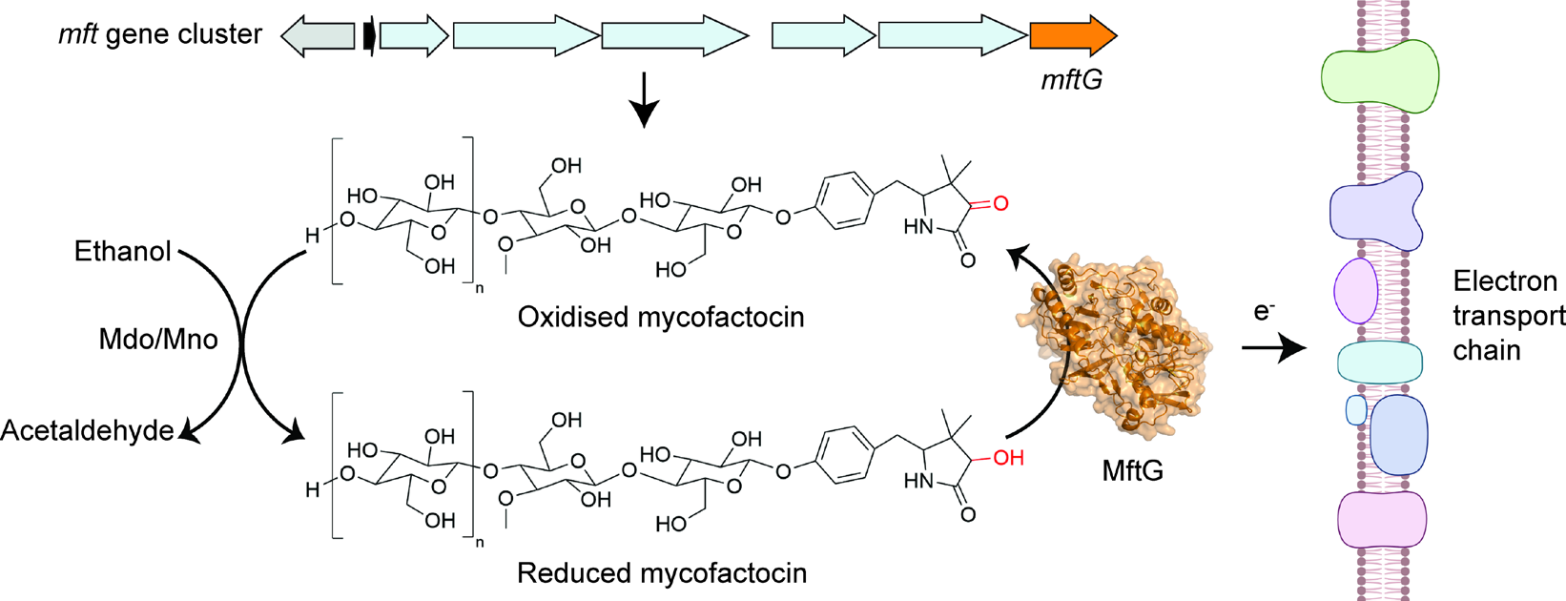
The enzyme MftG links ethanol metabolism to the electron transport chain. The cofactor mycofactocin, which is produced by the *mft* gene cluster (top arrows), links ethanol metabolism to the electron transport chain. This process begins when an enzyme known as Mdo/Mno catalyses the conversion of ethanol into acetaldehyde (left), transferring electrons to mycofactocin. Experiments by Graça et al. in *M. smegmatis* revealed that a previously uncharacterised gene in this cluster, known as *mftG* (orange arrow), encodes an enzyme (MftG; orange structure) that catalyses the transfer of electrons from the reduced mycofactocin to the electron transport chain. Electrons are passed along this chain of protein complexes in the mycobacterial membrane (right), which ultimately generates energy in the form of ATP. This process allows *M. smegmatis* to use ethanol as an energy source when other nutrients are not available. This figure was created with BioRender.com.

Image credit: Adapted from Figure 1 of Graça et al., 2025 (CC BY 4.0). The AlphaFold (Jumper et al., 2021) structure prediction of MftG was retrieved from the AlphaFold Protein Structure Database (Varadi et al., 2022) and visualised using PyMOL (Schrödinger, LLC, 2024).

Based on these findings, Graça et al. propose a model in which MftG regenerates mycofactocin by catalysing the transfer of electrons to the electron transport chain. In this model, MftG functions alongside the enzyme Mdo/Mno (which catalyses the initial transfer of electrons from ethanol to mycofactocin) to shuttle electrons from alcohol substrates to the electron transport chain. As Graça et al. note, a key question for future research is which specific component(s) of the electron transport chain MftG interacts with and delivers electrons to. In summary, Graça et al. have demonstrated an important new role for the mycofactocin cofactor in supporting the metabolic adaptability of mycobacterial cells under complex environmental conditions.
